# Supplementary material for: Circulating tumour DNA from the cerebrospinal fluid allows the characterisation and monitoring of medulloblastoma
Source: Nat Commun. 2020 Oct 27;11:5376. doi: 10.1038/s41467-020-19175-0 (PMC7591522; doi:10.1038/s41467-020-19175-0)
Supplement: Supplementary file 1 — Supplementary Information [file 41467_2020_19175_MOESM1_ESM.pdf]

Circulating tumour DNA from the cerebrospinal fluid allows the characterisation and monitoring of medulloblastoma

Escudero *et al.*,

SUPPLEMENTARY INFORMATION

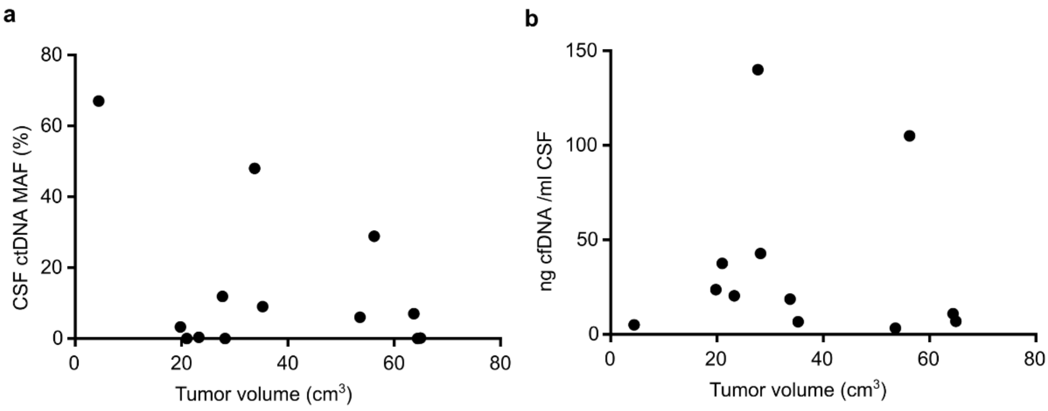

Supplementary Figure 1 | Correlation of tumor volume (cm^3) with (a) CSF ctDNA VAF (%) and (b) cfDNA (ng/ml). CSF samples were collected before the surgical resection for the 13 patients. CSF ctDNA MAF (%) analysed by ddPCR as previously shown in Fig. 2a for the 13 patients. The concentrations of CSF cfDNA (ng/ml) were calculated from 12 patients. No correlations were identified (Spearman r, two-tailed, p=0.5634 and p= 0.5731 respectively).

VAF (%): 100, 80, 60, 40, 20, 0

a MB6 SRCAP:p.R2444*

Time (days)	ddPCR validation (VAF%) Tumour	CSF	Plasma
0		48	ND
6	44	–	–
580		ND	ND
854		ND	ND
911		ND	–
975		ND	ND
1064		*	–
1076		–	ND
1281		ND	–
1286		0.2	–

b MB6 GJC3:p.T55I

Time (days)	ddPCR validation (VAF%) Tumour	CSF	Plasma
0		14	ND
6	ND	–	–
580		ND	ND
854		ND	ND
911		ND	–
975		ND	ND
1064		*	–
1076		–	ND
1281		0.1	–
1286		ND	–

c MB13 BMP5:p.R321*

Time (days)	ddPCR validation (VAF%) Tumour	CSF	Plasma
0		28.9	ND
3	48	–	–
16		7.6	ND
120		*	–
128		1.1	–
342		ND	ND

d MB13 MIB1:p.M468I

Time (days)	ddPCR validation (VAF%) Tumour	CSF	Plasma
0		12.1	ND
3	32	–	–
16		2.9	ND
120		*	–
128		ND	–
342		ND	ND

e MB8 PTCH1:p.L1024Pfs

Time (days)	ddPCR validation (VAF%) Tumour	CSF	Plasma
1115	93.4	57	ND

f MB8 KMT2A:p.W1199S

Time (days)	ddPCR validation (VAF%) Tumour	CSF	Plasma
1115	ND	35	ND

g MB8 CDKN2B:p.G124D

Time (days)	ddPCR validation (VAF%) Tumour	CSF	Plasma
1115	45.7	38	ND

h MB5 HCN1:p.Q828*

Time (days)	ddPCR validation (VAF%) Tumour	CSF	Plasma
0	48.5	7.6	ND
910	48	–	–
1092		0	–
1160		1	–
1166		0	ND
1168		0	–
1173		0.7	–

i MB5 ADD1:p.P718L

Time (days)	ddPCR validation (VAF%) Tumour	CSF	Plasma
0	ND	*	ND
910	1.8	–	–
1092		41.6	–
1160		48	–
1166		36	ND
1168		42.4	–
1173		52	–

j MB5 ACTA:p.Y190S

Time (days)	ddPCR validation (VAF%) Tumour	CSF	Plasma
0	ND	*	ND
910	0.9	–	–
1092		13.2	–
1160		13.3	–
1166		16.1	ND
1168		13.8	–
1173		15	–

Supplementary Figure 2 | Selected mutations identified from the WES analysis of 4 MB patients (MB5, MB6, MB8 and MB13) were validated using ddPCR in tumour/normal DNA, and follow-up CSF and plasma cfDNA samples. CSF ctDNA VAFs highlighted with a black box correspond to the samples used for WES (CSF1, CSF2). * indicates that all CSF cfDNA was used for WES.

Supplementary Table 1 | Classification

Schwalbe et al., 2017

Mutations						
WNT	SHH-child	SHH-infant	Grp4-HR	Grp4-LR	Grp3-LR	Grp3-HR
TP53	TP53	SUFU				GFI1
CTNNB1	TP53 GL	PTCH1				
	TERT					
	SUFU					
	PTCH1					
**Cytogenetics**						
WNT	SHH-child	SHH-infant	Grp4-HR	Grp4-LR	Grp3-LR	Grp3-HR
6-	9p+	9q-	i17q	17p+	7+	5+
	17p-			7+	14+	8+
	9q-			18+	18+	13+
	MYCN amp			i17q	4-	i17q
	GLI2 amp			8-	8-	16q-
				11-	10-	MYC amp
				16q-	11-	
				MYCN amp	13-	
					15-	
					16-	
					21-	

Hovestadt et al., 2020

Mutations												
WNT	SHH				Group3 / Group4							
	α	β	γ	δ	I	II	III	IV	V	VI	VII	VIII
CTNNB1	TP53	PTCH1	PTCH1	PTCH1	GFI1 act	GFI1 act				PRDM6 act	KBTBD4	PRDM6 act
DDX3X	PTCH1 (less)	KMT2D	SMO	TERT promoter	GFI1B act	GFI1B act						KDM6A
SMARCA4		SUFU	BCOR			KBTBD4						ZMYM3
						CTDNEP1						KMT2C
						SMARCA4						
						KMT2D						
**Cytogenetics**												
WNT	SHH				Group3 / Group4							
	α	β	γ	δ	I	II	III	IV	V	VI	VII	VIII
6-	9p+	2+	9q-	9q-	OTX2 amp	8+	7+	14+	7+	7+	7+	i17q
	9q-	SUFU del	PTEN del	14q-		1q+	10q-	7+	16q-	8-	8-	
	10q-	PTEN del				MYC amp	MYC amp	8-	MYC amp	11-	i17q (less)	
	17p-					i17q (less)	i17q	10-	MYCN amp	MYCN amp (less)		
	MYCN amp							11-	i17q	i17q		
	GLI2 amp							16-				

	Schwalbe et al., 2017							Hovestadt et al., 2020												
								WNT	SHH				Group3							Group4
PATIENT_ID	WNT	SHH-child	SHH-infant	Grp4-HR	Grp4-LR	Grp3-LR	Grp3-HR		α	β	γ	δ	I	II	III	IV	V	VI	VII	VIII
MB1	-	-	-	-	-	-	-	-	-	-	-	-	-	-	-	-	-	-	-	-
MB2	-	-	-	-	7+	7+	-	-	-	-	-	-	-	-	7+	7+	7+	7+	7+	-
MB3	-	-	-	-	-	-	MYC amp	-	-	-	-	-	-	MYC amp	MYC amp	-	MYC amp	-	-	-
MB4	-	-	-	-	7+; 16q-	7+	-	-	-	-	-	-	-	-	7+	7+	7+; 16q-	7+	7+	-
MB5	-	TP53 GL; MYCN amp; GLI2 amp; 17p-	-	-	MYCNamp; 16q-; 7+	7+	-	-	TP53 GL; MYCN amp; GLI2 amp; 17p-; 10q-	SUFUdel; 2+	-	-	-	-	7+; 10q-	7+	MYCNamp; 16q-	(MYCNamp); 7+	7+	-
MB6	-	17p-	-	-	-	-	-	-	17p-	-	-	-	-	-	-	-	-	-	-	-
MB7	-	17p-	-	-	7+; 11-	7+; 11-	-	-	17p-	-	-	-	-	-	-	7+; 11-	7+	7+; 11-	-	-
MB8	-	PTCH1; TP53; MYCNamp; 17p-; 9q-	-	-	8-	8-	-	-	PTCH1; TP53; MYCNamp; 17p-; 9q-	PTCH1	9q-	-	-	-	-	8-	-	8-	8-	-
MB9	CTNNB1; 6-	17p-	-	-	-	-	-	CTNNB1; 6-	17p-	-	-	-	-	-	-	-	-	-	-	-
MB10	-	-	-	-	-	-	-	-	-	-	-	-	-	-	-	-	-	-	-	-
MB11	-	17p-	-	-	-	-	-	-	17p-	-	-	-	-	-	-	-	-	-	-	-
MB12	-	PTCH1; MYCN amp; GLI2 amp	-	-	-	-	-	-	PTCH1; MYCN amp; GLI2 amp; 10q-	PTCH1; KMT2D; PTEN del	PTCH1; BCOR; PTEN del	-	-	-	10q-	-	-	-	-	-
MB13	-	TP53 GL; GLI2 amp	-	-	11-	10- ;11-	-	-	TP53 GL; GLI2 amp	-	-	14q-	-	-	-	10- ;11-	-	11-	-	-
MB5CSF	-	TP53 GL; MYCN amp; GLI2 amp; 17p- (33%)	-	-	MYCNamp	7+	-	-	TP53 GL; MYCN amp; GLI2 amp; 17p- (33%)	-	-	-	-	-	-	-	MYCNamp	(MYCNamp)	-	-
MB6CSF	-	17p-	-	-	-	-	-	-	17p-	-	-	-	-	-	-	-	-	-	-	-
MB8CSF	-	PTCH1; TP53; MYCNamp; 17p-; 9q-	-	-	8-	8-	-	-	PTCH1; TP53; MYCNamp; 17p-; 9q-	PTCH1	9q-	-	OTX2amp	-	-	8-	-	8-	8-	-
MB13CSF	-	TP53 GL; GLI2 amp	-	-	11-	10- ;11-	-	-	TP53 GL; GLI2 amp	-	-	14q-	-	-	-	10- ;11-	-	11-	-	-

Supplementary Table 2. Patient monitoring and ctDNA dynamics for 9 MB patients

PATIENT_ID	MUTATION	TIME (Days)	TUMOUR_VAF (%)	PLASMA_VAF (%)	CSF_VAF (%)
MB1	RIOK2:p.S456L	0	-	ND	7
		6	47	-	-
		487	-	-	ND
MB2	PUS7:p.D535A	0	15,9	ND	3,3
		1431	-	ND	ND
MB3	TRRAP:p.E3107K	0	63	ND	11,9
		1414	-	ND	ND
MB4	XRCC5:p.K35N	0	50	ND	0,38
		527	-	-	ND
		1347	-	ND	ND
MB7	YY1AP1:p.R665*	0	29	ND	0,18
		744		ND	ND
MB9	CTNNB1:p.S37C	0	47,2	2,2	ND
		525	-	ND	ND
MB10	HSPG2:p.R3992H	0	45	ND	ND
		476	-	-	ND
MB11	NFIA:p.R322H	0	15,5	ND	9
		394	ND	ND	ND
MB12	BCOR:p.E518*	0 (biopsy)	97,9	-	-
		240	-	ND	ND
		652	-	-	ND
