# Supplementary material for: Circulating tumour DNA from the cerebrospinal fluid allows the characterisation and monitoring of medulloblastoma
Source: Nat Commun. 2020 Oct 27;11:5376. doi: 10.1038/s41467-020-19175-0 (PMC7591522; doi:10.1038/s41467-020-19175-0)
Supplement: Supplementary file 3 — Description of Additional Supplementary Files [file 41467_2020_19175_MOESM3_ESM.pdf]

Description of Additional Supplementary Files

File Name: Supplementary Data 1
Description: Alignment information from the WES

File Name: Supplementary Data 2
Description: Germline mutations

File Name: Supplementary Data 3
Description: List of mutations identified T/N

File Name: Supplementary Data 4
Description: List of mutations MB5 T/N/CSF0/CSF1173

File Name: Supplementary Data 5
Description: List of mutations MB6 T/N/CSF0/CSF1064

File Name: Supplementary Data 6
Description: List of mutations MB8 T/N/CSF1115

File Name: Supplementary Data 7
Description: List of mutations MB13 T/N/CSF0/CSF120
