# Supplementary material for: Circulating tumour DNA from the cerebrospinal fluid allows the characterisation and monitoring of medulloblastoma
Source: Nat Commun. 2020 Oct 27;11:5376. doi: 10.1038/s41467-020-19175-0 (PMC7591522; doi:10.1038/s41467-020-19175-0)
Supplement: Supplementary file 11 — Reporting Summary [file 41467_2020_19175_MOESM11_ESM.pdf]

nature research

Corresponding author(s): Joan Seoane

Last updated by author(s): 30/9/20

Reporting Summary

Nature Research wishes to improve the reproducibility of the work that we publish. This form provides structure for consistency and transparency in reporting. For further information on Nature Research policies, see our Editorial Policies and the Editorial Policy Checklist.

Statistics

For all statistical analyses, confirm that the following items are present in the figure legend, table legend, main text, or Methods section.

n/a	Confirmed	
☐	☒	The exact sample size (n) for each experimental group/condition, given as a discrete number and unit of measurement
☐	☒	A statement on whether measurements were taken from distinct samples or whether the same sample was measured repeatedly
☐	☒	The statistical test(s) used AND whether they are one- or two-sided<br>*Only common tests should be described solely by name; describe more complex techniques in the Methods section.*
☐	☒	A description of all covariates tested
☐	☒	A description of any assumptions or corrections, such as tests of normality and adjustment for multiple comparisons
☐	☒	A full description of the statistical parameters including central tendency (e.g. means) or other basic estimates (e.g. regression coefficient) AND variation (e.g. standard deviation) or associated estimates of uncertainty (e.g. confidence intervals)
☐	☒	For null hypothesis testing, the test statistic (e.g. F, t, r) with confidence intervals, effect sizes, degrees of freedom and P value noted<br>*Give P values as exact values whenever suitable.*
☒	☐	For Bayesian analysis, information on the choice of priors and Markov chain Monte Carlo settings
☒	☐	For hierarchical and complex designs, identification of the appropriate level for tests and full reporting of outcomes
☐	☒	Estimates of effect sizes (e.g. Cohen's d, Pearson's r), indicating how they were calculated

Our web collection on statistics for biologists contains articles on many of the points above.

Software and code

Policy information about availability of computer code

Data collection	No software was used to collect data
Data analysis	DNA sequencing processing and analysis was performed using using BWA-MEM, Sidrón, BCFTOOLS, exome2cnv as described in the methods section.

For manuscripts utilizing custom algorithms or software that are central to the research but not yet described in published literature, software must be made available to editors and reviewers. We strongly encourage code deposition in a community repository (e.g. GitHub). See the Nature Research guidelines for submitting code & software for further information.

Data

Policy information about availability of data

All manuscripts must include a data availability statement. This statement should provide the following information, where applicable:

- Accession codes, unique identifiers, or web links for publicly available datasets
- A list of figures that have associated raw data
- A description of any restrictions on data availability

The DNA sequencing data have been deposited in the European Genome-phenome Archive (EGA) database under the accession code EGAS00001004651 [https://www.ebi.ac.uk/ega/studies/EGAS00001004651]. The data underlying Figs 1-5 and Supplementary Table 1 are provided as Supplementary Data 1-7. All the other data supporting the findings of this study are available within the article and its supplementary information files and from the corresponding author upon reasonable request. A reporting summary for this article is available as a Supplementary Information file.

Field-specific reporting

Please select the one below that is the best fit for your research. If you are not sure, read the appropriate sections before making your selection.

☒ Life sciences ☐ Behavioural & social sciences ☐ Ecological, evolutionary & environmental sciences

For a reference copy of the document with all sections, see nature.com/documents/nr-reporting-summary-flat.pdf

Life sciences study design

All studies must disclose on these points even when the disclosure is negative.

Sample size	A total of 13 paediatric patients diagnosed with medulloblastoma were included in the study. All patients were diagnosed and treated at the Vall d'Hebron University Hospital (Barcelona, Spain).
Data exclusions	No data was excluded for the analysis.
Replication	Tumour, normal and CSF samples were sequenced and the results obtained from the analysis of tumour and CSF samples were compared.
Randomization	This is not relevant to the study, patients were not allocated into experimental groups. Samples from 13 patients were collected and analysis was performed.
Blinding	This is not relevant to the study. Samples from 13 patients were collected and analysis was performed following the same protocol for all samples, as described in the methods section.

Reporting for specific materials, systems and methods

We require information from authors about some types of materials, experimental systems and methods used in many studies. Here, indicate whether each material, system or method listed is relevant to your study. If you are not sure if a list item applies to your research, read the appropriate section before selecting a response.

Materials & experimental systems

n/a	Involved in the study	
☒	☐	Antibodies
☒	☐	Eukaryotic cell lines
☒	☐	Palaeontology and archaeology
☒	☐	Animals and other organisms
☐	☒	Human research participants
☒	☐	Clinical data
☒	☐	Dual use research of concern

Methods

n/a	Involved in the study	
☒	☐	ChIP-seq
☒	☐	Flow cytometry
☒	☐	MRI-based neuroimaging

Human research participants

Policy information about studies involving human research participants

Population characteristics	Information about the characteristics of the cohort of 13 paediatric patients diagnosed with medulloblastoma including age (0-14 years old), Sex (6 males, 7 females) ,and clinical features is included in Figure 1.
Recruitment	All 13 paediatric patients diagnosed with medulloblastoma were diagnosed and treated at the Vall d'Hebron University Hospital (Barcelona, Spain).
Ethics oversight	A written consent, indicating that the samples obtained as standard of care in the clinical practice could be used for research, was obtained from the parents/legal representant in agreement with the declaration of Helsinki. The study was approved by the local IRB.

Note that full information on the approval of the study protocol must also be provided in the manuscript.
